# Augmenting plant-pollinator interactions to promote biodiversity and global food security

**DOI:** 10.3389/fpls.2026.1761150

**Published:** 2026-04-15

**Authors:** Sangam L. Dwivedi, Vincent A. Ricigliano, Giuseppe Forlani, Rodomiro Ortiz

**Affiliations:** 1 Independent Researcher, Hyderabad, India; 2 Invasive Species and Pollinator Health Research Unit, USDA-ARS, Davis, CA, United States; 3 Department of Life Science and Biotechnology, University of Ferrara, Ferrara, Italy; 4 Department of Plant Breeding, Swedish University of Agricultural Science, Alnarp, Sweden

**Keywords:** domestication, hybrid vigor, inbreeding, mating systems, plant-pollinator interactions, pollinator-relevant functional traits, polyploidy, secondary metabolites

## Abstract

Global agricultural production is currently limited by ongoing climate change. Approximately 90% of crop species and numerous wild plants are dependent on pollinators for reproduction. The global threat to pollinators posed by climate change has grown considerably, as higher temperatures, shifting rainfall patterns, and more frequent extreme weather events disrupt the fragile relationships between plants and their pollinators. The decline in pollinators is also linked to shifts in land use, the widespread adoption of monocropping, and heavy reliance on agrochemicals. Therefore, the protection of pollinators and the preservation of agrobiodiversity are essential to uphold global food systems. Here, we synthesize the adverse impact of climate change on plant-pollinator interactions; throughput assay for phenotyping floral traits; assessing variability and molecular basis of floral display (flower size, shape, color, attractants etc.) and reward (nectar volume and composition, pollen, and fragrance in case of ornamental plants) traits; crop domestication and inbreeding, ploidy and mating systems differences impacting plant-pollinator interactions; volatiles and metabolites mediating plant-pollinator relationships; trade-offs involving reproductive and pollinator traits; and finally, progress in developing pollinator-friendly crop cultivars through conventional plant breeding and biotechnological interventions. Pollinator-assisted phenotyping and selection platform (DARkWIN) combined with other high-throughput phenotyping assays, has the potential to simultaneously quantify multiple interactions impacting pollinators’ visitation and foraging behaviors, and generate data on other parameters like stress tolerance, yield, and nutrition in the target populations. Assessing and exploiting functional diversity for plant-pollinator interactions, combined with the use of functionally characterized genes and associated markers for floral display (*AT2G31010*, *AT4G17080*, *CmGEG*, *CmCYC2c*, *CmJAZ1-like-CmBPE2*, *Cyc2CL-1*, *Cyc2CL-2*) and reward (*SWEET9*, *BrCWINV4A*, *EOBI*, *EOBII*) traits, can be deployed in breeding programs to develop pollinator-friendly crop cultivars. Numerous candidate genes, reported herein, must be functionally validated before being deployed in crop breeding programs.

## Climate change effects on plant-pollinator interactions and crop quality

Climate change has emerged as a significant threat to pollinators worldwide, with profound implications for biodiversity and food security. Rising temperatures, altered precipitation patterns, and increased frequency of extreme weather events disrupt the delicate balance of plant-pollinator interactions. These changes affect the stability and resilience of pollination services, which are essential for the reproduction of over 75% of crop species and countless wild plants (Klein et al., 2007, Settele et al., 2016), thereby linking human food security to that of pollinators (Frigero et al., 2025). Temperature and water stress, alone or in combination, reduce the quantity and quality of floral nectar and pollen. Nectar volume in bee-pollinated species of Borago (*Borago officinalis*), an annual plant native to the Mediterranean region, decreased substantially with temperature rise and water stress, resulting in a 60% decrease in the total quantity of nectar sugars per flower. Temperature rise (and not water stress) reduced pollen weight per flower by 50% but increased pollen peptide concentration by 65%. The high temperature and water stress together increased total amino acid concentration and essential amino acid (%) in nectar but not in pollen. The combined stress also modified the relative % of amino acids in pollen and nectar (Descamps et al., 2021a). High air temperature may affect plant-pollinator interactions. Exposure of *B. officinalis* to elevated air temperature during flowering negatively affected floral signals (display and flower size) and floral reward (nectar and pollen) traits by altering bumblebee (*Bombus terrestris*) visitation and foraging behavior. *B. officinalis* plants grown at 26 °C received four times fewer bumblebee visits than plants grown at 21 °C. Thus, an increase in air temperature could reduce plant-pollinator interactions and reproductive fitness by changing flower visitation and foraging (Descamps et al., 2021b).

Temperature and irrigation, which affect pollination-mediated reproduction, act independently of one another in squash (*Cucurbita pepo*). The elevated air temperature decreased flower size and increased pollen production. The effect of soil-moisture limitation was uniformly inhibitory. The treatments did not change squash bee (*Xenoglossa* spp.) behavior. The floral visitation by the honeybee (*Apis mellifera*), however, increased with temperature in male flowers, while it decreased with soil moisture in female flowers. Though pollen deposition by bees was independent of plant soil moisture, the water stress increased pollen limitation. All these developments suggest that the transfer of lower-quality pollen from plants experiencing soil-moisture limitation led to drought-induced pollen limitation, thereby adversely impacting drought-induced plant-pollinator interactions (Gambel and Holway, 2024), supporting latter observations of drought stress effect on two flower sexes in monoecious Styrian oil pumpkin, *Cucurbita pepo* L. subsp. *pepo* var. *styriaca* Greb (Barman et al., 2024).

Assessing high temperature effects on foraging behaviors of the bumblebee (*Bombus terrestris*) in climate-controlled rooms revealed that the flower visitation time at 32 °C relative to 24 °C decreased while flower visitation rate and flight speed increased. It was consistent with the reduction in flight metabolic rate between the two temperatures. The number of trips made by each worker at 32 °C decreased, thus suggesting that, despite the reduced energetic cost, flight in elevated temperatures was stressful. Thus, elevated temperatures affect bumblebee foraging behavior, adversely disrupting plant-pollinator interactions (Gérard et al., 2024). A short exposure of flowering plants (blueberry [*Vaccinium corymbosum*], phacelia [*Phacelia tanacetifolia*] and white clover [*Trifolium repens*]) to extreme heat stress (37.5 °C vs normal temperature of 25 °C) for four hours under a no-choice field cage test during early bloom has indirectly affected bee (*Osmia lignaria*) and their offspring, with serious implications for crop pollination and native bee populations (Walters et al., 2025). High temperatures also affect the bumblebees’ ability to detect floral scents. Heatwaves induce strong reductions in *B. terrestris* antennal responses to floral scents. The reductions are generally stronger in workers than in males. However, bumblebees had no consistent pattern of recovery 24 h after heat stress. Thus, heatwaves may jeopardize bumblebee-mediated pollination services by disrupting the chemical communication between plants and pollinators (Nooten et al., 2024). However, efforts should be made to replicate these observations, focusing on the adverse effects of heat stress on visitation and foraging in field-grown crops.

Elevated temperatures, drought, and erratic weather can disrupt hive thermoregulation, reduce forage quality and availability, weaken immune responses, and exacerbate disease and parasite pressures (Zapata-Hernández et al., 2024). Global studies reveal that climate variables like heat and precipitation negatively affect honey bee food stores, mortality, metabolism, and pollination efficacy (Ostwald et al., 2024). However, current research is skewed toward short-term, small-scale experiments, with limited understanding of colony-level dynamics and/or long-term landscape-scale impacts. Despite the growing recognition of these threats, climate adaptation remains underrepresented in pollinator conservation strategies, which continue to prioritize land-use interventions without addressing the need for ecological connectivity or climate-resilient habitat design. The convergence of climate change with other anthropogenic stressors—such as pesticide exposure, invasive species, and monoculture farming—creates synergistic pressures that amplify pollinator declines (Marshman et al., 2019).

Elevated carbon dioxide (eCO_2_) can affect both plant growth and physiology, with the potential to alter pollen or plant tissue nutrition. Quantifying the effects of eCO_2_ on plant growth and pollen chemistry revealed that eCO_2_ is unlikely to uniformly change pollen chemistry or plant growth across multiple flowering plant species. However, it has the potential to alter ecological interactions, including its effect on specialized pollinators (Bernauer et al., 2024). eCO_2_ levels enhance flower visitation by buff-tailed bumblebee (*Bambus terrestris*) and European orchard bee (*Osmia cornuta*). At the same time, an increase in ozone (O_3_) led to a decline in visitation rates in faba bean (*Vicia faba*). The two greenhouse gases (GHGs) also showed an interactive effect on seed set in plants visited by *B. terrestris*. While eCO_2_ enhanced seed set, increased O_3_ decreased seed set, offsetting the positive effect of elevated CO_2_ on seed yield (Otieno et al., 2022). eCO_2_ and O_3_ individually and interactively alter emission of volatile organic compounds (VOC), nectar production in extra floral nectaries (EFN), and EFN visitation by the European orchard bee in the faba bean plant. While O_3_ exposure is linked with reduced nectar volume and a negative impact on EFN visitation by bees, eCO_2_ level had a positive impact on bee visits (Otieno et al., 2023). Plants produce volatile organic compounds (VOCs) emitted in the environment to attract pollinators, sensed mainly by their antennae. Exposure to high concentrations of O_3_ [0 ppb (control group), 80 ppb, 120 ppb, and 200 ppb] alters the emission of floral VOCs by plants, which persist in the atmosphere for the lifetime of pollinators, potentially impacting plant-pollinator interactions. The antennal activity of the honeybee (Genus *Apis)* is altered, depending on VOC structure and O3 concentrations. Exposure to O_3_ (80 ppb) decreases the superoxide dismutase enzyme activity rate. Clearly, demonstrating that air pollutants are harmful to pollinators, and may introduce bias in determining plant-insect interactions (Démares et al., 2024). Pollutants such as nitrate radicals (NO_3_) rapidly degrade floral scents, which potentially reduces pollinator attraction to flowers (Chan et al., 2024). Likewise, a high exposure of ozone (O_3_) concentration affected plant-pollinator interaction, as evidenced in the case of fig tree (*Ficus carica*) and its pollinator, the fig wasp (*Blastophaga psenes*) (Dubuisson et al., 2024). Air pollution may reduce the ability of pollinators to detect floral cues/odors, adversely impacting plant reproduction and pollinator fitness (Duque and Steffan-Dewenter, 2024).

**Figure 1:**
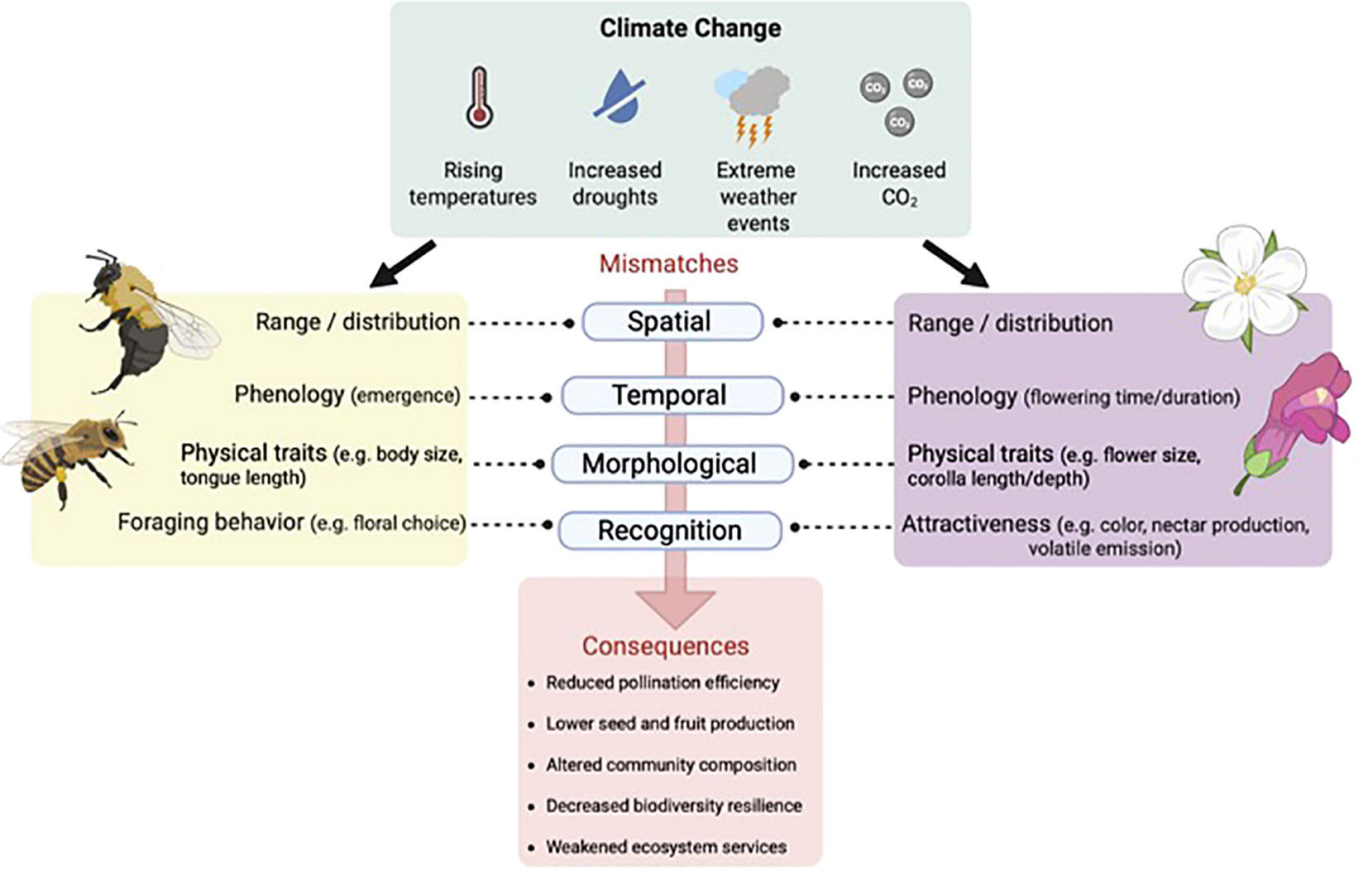
The top panel indicated key climate change drivers, including increased drought frequency, more extreme weather events, and elevated atmospheric CO_2_ levels. These factors influence a range of ecological and physiological processes in both plants and pollinators. As a result, mismatches can arise across four primary dimensions: (1) Spatial mismatches occur when plants and pollinators shift their geographic ranges at different rates or in different directions in response to changing environmental conditions, reducing their physical co-occurrence and interaction potential. (2) Temporal mismatches happen when flowering time and pollinator emergence become decoupled, often due to species-specific differences in phenological responses to temperature and precipitation cues. Even short-term misalignments can significantly reduce reproductive success. (3) Mechanical mismatches involve changes in physical traits—such as flower depth, plant height, or pollinator body size and tongue length—that impair effective pollen transfer. These mismatches are especially problematic in interactions requiring precise fit between floral and pollinator structures. (4) Sensory and behavioral mismatches result from altered floral signals (e.g. scent, color) or heat-induced impairments in pollinator foraging, learning, or cue recognition. These disruptions reduce pollinator attraction and visitation frequency, even when species co-occur. The combined effect of these mismatches weakens pollination efficiency, lowers seed and fruit production, and destabilizes plant–pollinator interaction networks. Over time, this can lead to shifts in community composition, declines in biodiversity, and reduced ecosystem resilience. While generalist species may buffer some of these disruptions through flexible behavior and broader partner ranges, specialized interactions are especially vulnerable.

Climate change drivers such as rising temperatures and altered precipitation may cause significant phenological shifts in plants and pollinators. As the climate warms, plants and pollinators respond at different rates, leading to mismatches (**Figure 1**) between flower availability and pollinator activity (de Manincor et al., 2023; Duchenne et al., 2020; Peng et al., 2025; Zoller et al., 2026). This decoupling may result in a mismatch between flowering plant and pollinator activity, negatively impacting pollination success and plant reproduction, thereby weakening interactions and reducing the fitness of both partners, leading to population declines (Fisogni et al., 2025). Even short-term warming may influence plant-pollinator interactions in many plant species by adversely affecting pollinator behavior and anthesis (Chang et al., 2024). While some generalist species may adapt by switching hosts, many specialist species, which form tightly synchronized mutualisms, face severe reproductive consequences (de Manincor et al., 2023; Kammerer et al., 2021; Weaver and Mallinger, 2022). An understanding of these complexities is therefore desirable to maintain phenological match for the persistence of plant-pollinator interactions, as well as to predict the consequences of climate-change-induced phenological mismatch for the development of strategies to mitigate their effects on plant-pollinator mutualisms.

Three-way interactions, involving pollinator species, pollen genotype, and plant cultivars, determine not only the yield of pollinator-dependent crops but also the nutritional quality (nutritional, sensory, and marketing value) of edible produce (Eilers et al., 2011; Ochola et al., 2025). For example, animal pollination increases fruit quality by 30% by enhancing organoleptic properties; pollinator species richness enhances impact minerals; visitation rate enhances macronutrients; and diverse pollinator traits optimize fruit quality (Ochola et al., 2025). Pollinators also support achieving sustainable development goals, for example, ‘zero hunger’ and ‘life on earth’ (Peixoto et al., 2022). A report indicates reduced food crop quality under inadequate animal pollination; therefore, safeguarding pollination services is important for maintaining food and nutritional security (Gazzea et al., 2023). Bee and non-bee insect pollinators play a major role in the quantity and quality of food crops (Requier et al., 2023). Understanding the mechanisms underlying crop pollination and pollinator health in unison across a range of taxa is therefore a ‘*win-win situation*’ for stakeholders in industry and conservation (Jones and Rader, 2022).

An exhaustive search was made to survey peer-reviewed and Scopus-indexed articles from the Web of Science database, covering publications from 2000 to date, to search for targeted literature in high-impact-factor journals using keywords and phrases to provide an in-depth synthesis of progress for the development of new germplasm favoring plant-pollinator interactions. The major focus is toward i) quantifying climate-induced alterations in pollinator-relevant floral resources impacting pollinator visitation, foraging behavior, and pollinator health, and on agrobiodiversity and food and nutritional security, ii) advances in pollinator-assisted phenotyping and selection platform to quantify multiple interactions impacting pollinators visitation and foraging behavior, iii) the presence of natural variability for pollinator-dependent floral display and reward traits in genepool, iv) genomic regions (QTL), candidate and functionally characterized genes and DNA markers associated with floral traits, vi) effect of crop domestication, ploidy, mating systems, and inbreeding impacting plant-pollinator interactions, vii) fragrance and metabolites mediating plant-pollinator relationships, viii) trade-offs involving reproductive and pollinator-dependent floral traits, and finally, ix) how this knowledge could be harnessed to develop pollinator-friendly crop germplasms, combining stress tolerance, productivity, and nutritional quality through conventional plant breeding and biotechnological interventions.

## Pollinators-assisted plant phenotyping for visitation and foraging

Until the development of ‘DARkWIN’ platform (Pérez-Alfocea et al., 2024), there were strong bottlenecks in quantifying flower traits to relate it to an accurate genotype-to-phenotype prediction for plant-pollinator interactions. DARkWIN leverages the natural interactions between plants and pollinators to evaluate plant quality and stress resistance (Pérez-Alfocea et al., 2024), thereby offering a more nuanced understanding of how different genotypes respond to various environmental conditions. Through this advanced insect geo-positioning system, the complex interactions between plants and their pollinators can be precisely traced and analyzed, thus providing valuable insights into visitation patterns and foraging behaviors. More recently, a five-step workflow have been recently proposed for optimizing pollination of entomophilous crops (i.e., crops that insects pollinate), which include (i) chemical phenotyping of flowers and floral rewards, (ii) chemical phenotyping of floral volatile organic compounds (VOCs), (iii) CT scans detailing morphological phenotyping of flower shapes, (iv) pollinator video tracking and behavioral analysis, and (v) data integration, GWAS, selection, and breeding. It effectively maps and selects genotypes with floral traits that actively guide pollinator preferences. These technological advances and automation facilitated high-throughput metabolic phenotyping of floral chemical traits of pollinator attraction and rewards. Integrating these measurements with CT scans of flower shape analysis and video tracking of pollinator behavior may guide the selection of segregants (or genotypes in genepool) with enhanced insect visitation rates and effective cross-pollination, as evidenced in the case of blueberry*, Vaccinium corymbosum*, a crop heavily dependent on bee pollination for fruit production (Borghi et al., 2025).

Plant physiology underpins the DARkWIN concept by considering the relation between organs producing photo assimilates (the source) and those consuming or storing them (the sink), integrating the supply of water and nutrients from the surrounding environment (e.g. soil and solar radiation) into crop productivity following plant growth, flowering, and fruiting. This platform operates on the principle that flowers serve as highly sensitive indicators of plant stress responses, with their chemical composition and resource production directly reflecting environmental pressures. This sensitivity makes pollinator preferences particularly valuable as natural selection markers, allowing researchers to identify plants with superior resilience characteristics. Through automated tracking systems, researchers can quantify how different genotypes influence pollinator attraction under varying environmental conditions, thus developing a comprehensive dataset of plant-pollinator interactions.

The integration of this technology with traditional phenotyping methods enables bridging the gap between genotype expression and ecological function. By analyzing pollinator behavior patterns across multiple environmental conditions, the understanding of how genetic traits manifest in real-world scenarios improves, thus guiding breeding for enhanced crop resilience. The platform’s ability to capture detailed behavioral data allows us to further validate the findings through metabolic, transcriptomic, and ionomic analyses, which facilitates establishing a robust framework for understanding plant-pollinator relationships.

## Breeding for pollinator-friendly traits in crops

### Natural variation associated in pollinator-relevant floral traits

The key to success in crop improvement depends on a continued supply of genetic diversity, including new or improved variability for target traits. Plants adopt a variety of means to attract pollinators for the transfer of pollen for reproduction. Pollinator-relevant floral traits are those that enhance their ability to visit and pollinate the flowers, for example, flower color, size, and shape, or corolla depth, while pollinator-relevant floral reward traits include nectar, pollen grains, or scent in *Lathyrus odoratus* (sweet peas) and *Nicotiana alata* (Jasmine or sweet tobacco) to attract specific groups of pollinators to visit flowers. The pollinators derive their dietary and energy requirements by feeding on nectar or pollen grains. The quantity (volume) and composition of nectar, particularly sugar composition, are the key determinants, influencing plant-pollinator interactions. Sucrose, glucose, and fructose are the predominant sugars in nectar, which may vary widely across species. The different pollinator taxa, such as honeybees (genus *Apis*) vs bumblebees (genus *Bombus*) vs solitary bees (*Osmia, Megachile, Xylocopa, Andrena genus*) prioritize various cues depending on morphology and foraging goals (Engel et al., 2021). Reports suggest sufficient genetic variability for pollinator-relevant floral traits (**Tables 1**, **2**) in diverse crops.

**Table 1:** Variability for pollinator-dependent floral traits (flower size and shape, color etc.) in germplasm pool.

Pollinator-dependent floral traits	Reference
Forage crops	
Alfalfa (*Medicago sativa*)	
Plants with more racemes per stem and more stems per plant; hue or flower color; chroma or darkness of flowers	Brunet et al., 2021
Grain crops	
Cowpea (*Vigna unguiculata*)	
Colored flower (white, purple, yellow), inflorescence position, hour flowers remained open during the day (FRODD)	Lazaridi et al., 2023
Faba bean (*Vicia faba*)	
Flower shape, petal size, corolla-tube length, and petal spot size	Bailes et al., 2023
Greater intensity of ‘streaks on standard petal’ and ‘extent of anthocyanin coloration on standard petal’ differentiated landrace (Episkopis) from modern cultivar (Histal)	Barda et al., 2023
Oil crop	
Mustard (*Brassica rapa*)	
OP, GMS, and CMS hybrid cultivars differed for size of flowers and total flowers per plant; GMS and CMS hybrids had greater petal size than OP cultivars	Carruthers et al., 2017
Soybean (*Glycine max*)	
Accessions with purple flower attracted more pollinators than white flower	Chacoff et al., 2024
Sunflower (*Helianthus annuus*)	
Display extreme diversity for patterns of ultraviolet pigmentation to pollinators	Todesco et al., 2022
Significant variability for flower morphological and color (petal carotenoids and disc anthocyanin) traits, with sufficient trait plasticity between environments	Dowell et al., 2019
Significant variability for floret lengths; floret length closely related to corolla depth during anthesis; floret size contributed 52% phenotypic variation in wild bee preference; a reduction of 2 mm in floret length more than doubled pollinator activity	Portlas et al., 2018
Significant differences in corolla length among inbred lines	Mallinger and Prasifka, 2017
Fruits crop	
Blueberry (*Vaccinium* spp.)	
Variability in flower density, corolla length and the length-to-width ratio	Cromie et al., 2024
Strawberry (*Fragaria × ananassa*)	
Significant variation for flower size and shape	Symington and Glover, 2024
Industrial crop	
Cotton (*Gossypium* sps.)	
Sufficient variation in pollinator-attracting purple spots (purple, pink, or blue colors)	Abid et al., 2023
Ornamental crop	
Chrysanthemum (*Chrysanthemum indicum*)	
Substantial variation in flower color types	Wang et al., 2025
Wide range of variations in flower color	Wan et al., 2024
Abundant variation in flower color	Song et al., 2023
*Petunia*: F7 RILs (*Petunia axillaris* × *Petunia exserta*)	
Sufficient variability for flower counts, flower diameter, flower length	
Velvet turnera *(Turner velutina*)	
Larger flowers	Ramos et al., 2025

**Table 2:** Variability for pollinator-dependent floral reward traits (nectary size and shape; pollen grain and nectar volume and composition) in germplasm pool.

Pollinator-dependent floral reward traits	Reference
Grain crops	
Cowpea (*Vigna unguiculata*)	
Nectar, rather than flower color, more attractive to pollinators	Dingha et al., 2021
Faba bean (*Vicia faba*)	
Floral volatile compounds	Bailes et al., 2023
Landrace (Episkopis) enriched with nectar compounds (amino acids and sugars) than modern cultivar (Histal)	Barda et al., 2023
Multiple folds differences in pollen production, nectar sugar concentration, nectar volume, and operative force to open *Vicia* flowers between lines	Bailes et al., 2018
Oil crop	
Mustard (*Brassica rapa*)	
GMS hybrid produced more per flower nectar and sugar than CMS or OP varieties; nectar production and amount of nectar sugar independent of number and size of flowers	Carruthers et al., 2017
Nectar sugar, pollen, and floral scent compound phenylacetaldehyde	Knauer and Schiestl, 2015
Rapeseed (*Brassica napus*)	
Nectar production (per hectare) but with similar sugar composition ratios and absolute sugar content in nectar	Na et al., 2024
Significant variability for solute concentrations, nectar volumes, sugars, and amino acids; up to 100 times higher amino acid concentration in phloem sap than in nectar	Bertazzini and Forlani, 2016
Sunflower (*Helianthus annuus*)	
Sufficient variability for nectar production among RILs	Barstow et al., 2022
Significant differences in the amount and composition of nectar sugars among inbred lines; Wild bee and honey bee visits significantly increased with nectar sugar amount and decreased with corolla length, however, unaffected by nectar sugar composition; sunflowers with greater quantities of nectar sugar and shorter corollas receive greater pollination services from both managed and wild bees	Mallinger and Prasifka, 2017
Fruit and vegetable crop	
Strawberry (*Fragaria × ananassa*)	
Significant variation for nectar and pollen production	Symington and Glover, 2024
Blueberry (*Vaccinium* spp.)	
Significant differences in nectar volume among accessions	Cromie et al., 2024
Pumpkin (*Cucurbita maxima* cv. Big Max)	
Differences in nectar composition between male and female flowers: 29 metabolites between male and female nectar; two female- and 10 male-specific unique proteins in nectar, respectively	Chatt et al., 2018
Ornamental crop	
Velvet turnera *(Turner velutina*)	
Sufficient variability in nectar sugar content	Ramos et al., 2025
Cleomaceae (Brassicales)	
Floral nectaries display Substantial diversity in floral nectaries size and shape, ranging from adaxial protrusions or concavities to annular disks	Zenchyzen et al., 2023

#### Floral display

Faba bean (*Vicia faba*) germplasm shows substantial variation in flower traits, with bees (*Bombus terrestris*) efficient in distinguishing between natural variation in petal spot size, floral volatile emissions, and corolla-tube length. The foragers prefer spotted flowers over non-spotted flowers, shorter corolla-tube length over longer tubes, and if paired with floral reward traits (fructose/glucose ratio, nectar (μL) per flower, sugar (μg) per flower, nectar (μL) per plant), it significantly enhances pollinator attraction to faba bean (Bailes et al., 2023). The flower streaks’ intensity, the degree of flower coloration, and nectar chemistry differentiated the faba bean landrace (Episkopis) from the modern cultivar (Histal). Landrace attracted more bees from different genera (*Apis*, *Anthophora*, *Eucera*) and bee visits compared to the modern cultivar, thereby suggesting that floral and nectar differences could be exploited for increased plant-pollination interactions in faba bean (Barda et al., 2023).

Many of the flower traits (i.e., color, inflorescence position, and the hours that flowers remained open during the day) promoted pollinator abundance and foraging among cowpea (*Vigna unguiculata*) accessions. These traits were, however, not linearly related to pollinators’ abundance and foraging; therefore, not suitable for selection aiming to increase pollinators’ visitation. Contrarily, floral reward traits were more important in attracting pollinators in cowpea (Dingha et al., 2021; Lazaridi et al., 2023). Accessions with purple flower color attracted more pollinators than those with white flowers in soybean, *Glycine max* (Chacoff et al., 2024). The operative force required to open faba bean flowers was significantly different between lines (Bailes et al., 2018). Open-pollinated (OP), genetic male sterility (GMS), and cytoplasmic male sterility (CMS) hybrid cultivars of mustard (*Brassica rapa*), under greenhouse evaluation, differed for flowers per plant and flower size (mm^2^), with GMS and CMS hybrid cultivars having greater petal size than OP cultivars (Carruthers et al., 2017). Wild sunflower inflorescences display extreme diversity for patterns of ultraviolet (UV) pigmentation to most pollinators (Todesco et al., 2022). Floret size (i.e., depth of the corolla) affects the accessibility of nectar in sunflowers (*Helianthus annuus*). The maintainer inbred lines show significant variation in floret size and its effect on pollinator visitation. The floret length ranged from 6.8 to 9.9 mm. It contributes 52% of the variation in wild bees’ preference. The floret length measured before anthesis is closely related to corolla depth during anthesis across environments. A reduction in floret length of 2 mm increases pollinator activity by more than double. Thus, inbreds and hybrids with smaller florets could enhance sunflower pollination (Portlas et al., 2018). Pima cotton (*Gossypium barbadense*) is characterized by a beauty mark (or spot) at the base of each petal, caused by a group of anthocyanin-pigmented cells. Contrarily, upland cotton (*G. hirsutum*) genotypes are devoid of this petal spot. The presence of beauty spots, ranging from purple, pink, or blue colors in cotton (*Gossypium barbadense*) flowers, attracts pollinators and protects plants from ultraviolet exposure (Abid et al., 2023). Such variability in floral display traits is also reported in a few fruits [blueberry (*Vaccinium* sect. *Cyanococcus*), strawberry (*Fragaria × ananassa*)] and ornamental [chrysanthemum (*Chrysanthemum indicum*), petunia (*Petunia* spp.), and velvet turnera (*Turnera velutina*)] plants (**Table 1**), with moderate to high heritability for selective breeding to breed new cultivars with attractive floral display for attracting pollinators.

#### Floral reward

Over 4-, 3-, and 39-fold differences in range variation were noted in pollen production, nectar sugar concentration, and nectar volume among faba bean germplasm, respectively (Bailes et al., 2018). Variability in nectar volume rather than variation in flower color attracted pollinators among cowpea germplasm (Dingha et al., 2021). Floral reward traits in greenhouse-grown mustard plants differed between open-pollinated (OP), genetic male sterility (GMS), and cytoplasmic male sterility (CMS) hybrid cultivars. The sugar concentration was consistent among and within the breeding systems. GMS hybrids, however, produced more nectar and more sugar per flower than CMS hybrids or OP cultivars. Nectar traits, except for a ratio of fructose/glucose in OP cultivars, were consistent within all breeding systems. GMS hybrids produced 1.73 times more nectar resource per plant than OP cultivars. Nectar production and sugar concentration in the nectar were independent of the number and size of flowers (Carruthers et al., 2017). Rapeseed (*Brassica napus*) cultivars differed significantly for nectar characteristics. The cultivars varied for absolute sugar content; however, sugar composition ratios were similar. The percentage of nectar amino acid composition remained relatively uniform, irrespective of the absolute concentrations. Per hectare production of honey from selected cultivars ranged from 107 to 42.4 kg ha^-1^ (Na et al., 2024). Pollen quantity and size in sunflowers are heritable traits and significantly correlated. Pollen quantity per floret is positively correlated with floret size, while floret size and pollen quantity are unrelated to pollen size. A strong and positive correlation between floret size and pollen quantity reveals a possible trade-off for pollinators. Wild bees prefer sunflowers with smaller florets (Prasifka et al., 2023a). US-bred sunflower inbred lines with greater nectar sugar (sucrose) and shorter corollas (petals) facilitate more bees’ visits (Mallinger and Prasifka, 2017). Biparental recombinant inbred lines (RILs), derived from lines with contrasting nectar volume, showed a large range variation for per floret nectar volume (μl), nectar sugars (μg) per floret, and nectar concentration (°Brix) (Barstow et al., 2022).

While most pumpkin (*Cucurbita* spp.) species are monoecious (i.e., male and female flowers on the same plant), a few others are dioecious species (i.e., male and female flowers on different plants). Autumn squash (*Cucurbita maxima* cv. Big Max] is a dioecious species. Natural variation in nectar volume and composition attracts bee species visiting winter squash. Metabolomic and proteomic assessments of the sex-dependent variation revealed 88 metabolites between male and female flowers, nectar, and the nectaries, of which 40 were positively identified. The identified metabolites belong to sugars, sugar alcohols, aromatics, diols, organic acids, and amino acids. Male and female flower nectar differ in 29 metabolites. About 70% of nectar protein overlapped between nectar types. Two and 10 unique proteins were associated with female- and male-specific nectars, respectively. Furthermore, the abundance of 45 proteins is significantly different between male and female nectaries, which may provide the targets to understand plant–pollinator interactions in winter squash (Chatt et al., 2018). Blueberry, strawberry, velvet turnera, and those from the *Cleomaceae* family (220 species and two genera: *Cleome* and *Cleomella*) showed significant variability affecting floral nectary size and shape, nectar or pollen production to attract pollinators (**Table 2**).

Conclusively, it shows variability in visitation and foraging behavior of pollinator preferences for floral and floral reward traits in pollinator-dependent crops. Identifying and exploiting such genetic variants from the genepool is the first step toward developing pollinator-relevant crops. Reduced subsets in the form of a core collection (Brown, 1989), representing the diversity of the entire collection of a given species preserved in the genebank, should be screened for variation in pollinator-relevant floral traits in crops that are predominantly pollinator dependent. Floral traits, like other quantitative traits (i.e., yield), are highly influenced by the genotype × environment interaction (Prasifka et al., 2023b). Hence, germplasm with distinct floral attributes should be evaluated across environments to identify accessions with distinct pollinator-favorable traits for use in crop improvement programs.

### Genetic architecture of floral display and reward traits

Sepals, petals, stamens, and pistils, that arise in four concentric rings or whorls, constitute a flower in plants. ABC model and MADS-box genes regulate flower organ development (Dwivedi et al., 2008, and references therein). Further, molecular and genetic analyses of floral morphogenesis and organ specification led to the ABCDE model of flower development in eudicots and grasses, while others appear to have unique and diversified functions (Ciaffi et al., 2011). Unlocking natural allelic variation associated with floral traits underlying pollinator specificity is critical to unfolding the molecular mechanisms governing plant-pollinator interactions. *Petunia*, *Mimulus* (monkeyflower), and *Antirrhinum* (dragon flower or snapdragon), among flowering plants, provided sufficient evidence to establish causal links from genes to floral traits to pollinator responses (Yuan et al., 2014).

### Functionally characterized genes

#### Floral display

The advantage of functionally characterized genes is that these can be deployed in breeding programs. For example, genes involved in petal width and short stamen length, *AT2G31010* and *AT4G17080*, in mustard, through hoverfly (*Eupeodes corollae*) pollination, could be used to shift mating systems from predominantly outcrossing to mixed mating or mustard as an inbreeder, if practiced long-term selection, as evidenced by a shift from outbreeder to mixed mating through hoverfly pollination over generations. Hoverfly pollination over several generations has led to genomic changes associated with a shift in the mating system from predominantly outcrossing to mixed mating (Kofler et al., 2024). Gene orthologs (*AT2G31010* and *AT4G17080*) could be used to change the mating system in those crops as well. Chrysanthemum is a highly favored ornamental plant known for its floral diversity and extensively investigated for genes associated with floral display traits. Elite genetic stocks could be tailored with diverse floral head types (*CmCYC2c*), petal growth and development (*CmGEG*, *CmJAZ1-like - CmBPE2*, *CmTCP20*, *Cyc2CL-1* and *Cyc2CL-2*) or for aborting stamens, and *PH4* floral pigmentation and scent emission (**Table 3**).

**Table 3:** Functionally characterized genes associated with floral display traits in chrysanthemum, mustard, and petunia.

Gene	Functional characterization	Reference
Mustard (*Brassica rapa*)		
*AT2G31010 and AT4G17080*	Involved in petal width and short stamen length	Kofler et al., 2024
Chrysanthemum (*Chrysanthemum* spp.)		
*CmGEG*	Inhibits petal growth	He et al., 2024
*CmCYC2c*	Diverse floral head types	Qiu et al., 2023
*CmJAZ1-like-CmBPE2*	Regulates petal size	Guan et al., 2022
*Cyc2CL-1 and Cyc2CL-2*	Regulates petal development and aborts stamens, respectively	Liu et al., 2021
*CmTCP20*	Regulates petal size	Wang et al., 2019
Petunia (*Petunia* spp.)		
*EOB1 and EOB2*	Regulates flower maturation and senescence	Chopy et al., 2023
*EOB1 and EOB2*	Mediates with floral scent emission	Yarahmadov et al., 2020
*PH4*	Integrates volatile production and emission processes, interconnecting flower color and scent	Can’ani et al., 2015

#### Floral reward

The nectar from *Brassica* species is rich in glucose and fructose, with very low levels of sucrose. Cell wall invertases (CWINVs) mediate the hydrolysis of sucrose into fructose and glucose. *BrCWINV4A* is predominantly expressed in *Brassica rapa* nectaries. The *brcwinv4a* mutant shows significantly reduced CWINVs activity in the nectaries, produces a sucrose-rich nectar, thereby demonstrating CWINVs activity is key to the production of fructose and glucose-rich nectar (Minami et al., 2021). Hence, *BrCWINV4A* could be deployed or its orthologs in other crops, to alter nectar composition from predominantly sucrose to fructose and glucose-rich nectar to attract pollinators for enhancing ecological adaptation and reproductive success in *Brassica* species. *SWEET9* functions as an efflux transporter and could be a potential gene to enhance nectar production and secretion in multiple species with gynecial nectaries (Lin et al., 2014), *PH4* volatile production and emission, while *EOB1* and *EOB2* could be deployed to regulate flower maturation and senescence or floral scent emission in the genus *Petunia* (**Table 3**).

### Genomic regions and putative genes

#### Floral display

**Table 4** lists genomic regions and/or putative genes associated with floral display traits in diverse crops. The petal length and expression levels of *HaMADS3*, *HaMADS7*, and *HaMADS8* in sunflower capitula are strongly correlated, while *HaCYC2c* and *HaYABBY* control petal elongation and floral symmetry (He et al., 2022; Wang et al., 2024a; Wu et al., 2020). By exploiting these genes in breeding programs, sunflower elite genetic stocks with diverse capitula, petal depth, and symmetry could be tailored to attract a specific group of pollinators. The *cis*-regulatory variation affecting *HaMYB111*, through accumulation of UV-absorbing flavonol glycosides in ligules (or petals) in wild sunflowers, shapes mutualistic interactions between plants and their pollinators, as well as abiotic selection pressures to influence floral diversity (Todesco et al., 2022). Ligules with larger UV-patterns could be exploited to enhance resistance to desiccation (i.e., a role in reducing water loss) in drier environments, thereby providing a dual role of floral UV patterns in pollinator attraction and abiotic response, suggesting a complex adaptive balance underlying the evolution of floral traits in sunflower (Todesco et al., 2022). Genome-wide association studies (GWAS)-based SNPs associated with floral morphology and carotenoids in sunflower showed substantial trait plasticity, with anthocyanin mapped more strongly than floral morphology. SNPs explained 16% to 24% of the variation in petal carotenoid content in field- and greenhouse-grown sunflowers, while disc size in the field environment explained up to 19% of phenotypic variation. Many of these loci lie within the genomic regions involved in sunflower domestication (Dowell et al., 2019). This difference in phenotypic expression between the two environments and SNP loci underlying the genomic regions involved in sunflower domestication may provide more opportunities to identify segregants for substantial trait plasticity in interspecific crosses with greater carotenoid (including anthocyanin) content in sunflower disc (or capitulum), providing intense flower color as a mechanism to attract pollinators.

**Table 4:** Quantitative trait loci (QTL) and candidate genes associated with floral display traits in diverse crop groups.

Genus	Candidate gene	Flower trait	Reference
Oil crop			
*Helianthus* (sunflower)	*HaMADS3*, *HaMADS7*, *HaMADS8*	Petal development	Wang et al., 2024
	*HaMYB111*	Ultraviolet pigmentation	Todesco et al., 2022
	SNPs across 10 linkage groups	Morphological (disk size) and color trait (petal carotenoid and anthocyanin) variation, with substantial trait plasticity	Dowell et al., 2019
Industrial crop			
*Gossypium* (cotton)	A single SNP (C/T) within the BM coding sequence	Associated with differences in beauty spots (purple, pink, or blue)	Abid et al., 2023
	*GbBM* (a semidominant gene, *Beauty Mark* (*BM*)	Regulates petal spot development	Abid et al., 2022
	*HaYABBY*	Petal elongation and floral symmetry	Wu et al., 2020
	*HaMYB111*	UV-absorbing flavonol glycosides in ligules	Todesco et al., 2022
	SNPs across 10 LGs	Significantly associated with floral morphology and color traits	Dowell et al., 2019
Ornamental crop			
*Chrysanthemum*	809 candidate genes, including *MYB, bHLH, WD40, NAC*, and *AP2/ERF* TFs, as well as cytochrome P450-type enzyme genes	Flower color	Wan et al., 2024
	1104 MYBs gene family, grouped into four subfamilies and 35 lineages, with whole-genome duplication and tandem duplication as major driving source of duplicates in *CmMYBs*; a few R2R3 MYBs mostly located on the distal telomere segments of the chromosomes subjected to positive selection; *CmMYBS2*, *CmMYB96*, and *CmMYB109* act as negative regulators for anthocyanin biosynthesis	Flower color	Wang et al., 2024
	3- and 2-major QTL associated with total anthocyanin and carotenoid contents, respectively; 17 unigenes in *Chrysanthemum nankingense* genome	Anthocyanin and carotenoids in flower	Song et al., 2023
	*CmCCD4a*	Petal color	Sumitomo et al., 2019
*Gerbera*	*GhBPE-GhPRGL*	Ray petal length	Jiang et al., 2022
	*GhMIF*	Petal elongation	Han et al., 2017
*Rhytidophyllum*	*RADIALIS*, *GLOBOSA*, *JAGGED*	*Corolla shape*	Poulin et al., 2022
*Petunia*	A single mutation of large phenotypic effect in *MYB-FL*	UV-absorbing flavonol pigments	Lüthi et al., 2022
	Major QTL	Flower length (35.5% PVE) and diameter (47.1% PVE)	Cao et al., 2019
	2–4 QTL	Associated with flower diameter, length, and count, with qFD3.1and qFD4.1 for flower diameter, qFL1.1 for flower length, and qFC2.1 and qFC4.1 for total flower count detected in both years	Cao et al., 2018
	Mutations in MYB-FL regulates transitions in UV absorbance	Gain of UV absorbance controls transition from bee to moth pollination, determined by a cis-regulatory mutation, while a frameshift mutation causes loss of UV absorbance during the transition from moth to hummingbird pollination	Sheehan et al., 2016
	*AN2*	Flower color	Hoballah et al., 2007

Hybrids are preferred over inbred cultivars due to increased biomass yields. The low efficiency and high cost of hybrid seed production limit the exploitation of heterosis in cotton. A semidominant gene, *Beauty Mark* (*GbBM*), regulates purple spot formation at the base of floral petals in the cultivated tetraploid cotton, *Gossypium barbadense*. The upland cotton (*G. hirsutum*) genotypes are devoid of this petal spot. *GbBM* targets four flavonoid biosynthesis genes that positively regulate petal spot development. The introgression of a *GbBM* allele into *G. hirsutum* restored petal spot formation, substantially increased the frequency of honeybee visits, and thereby improved cotton seed yield in a three-line hybrid production system (Abid et al., 2022). Hence, overcoming a major constraint in low seed production in elite cotton hybrid lines. Abid et al. (2023) further report that natural variation in Beauty mark is also associated with ultraviolet (UV)-based geographical adaptation in cotton species. Cotton plants with purple, pink, or blue color beauty spot attract pollinators, protect plants from ultraviolet (UV) radiation, and scavenge reactive oxygen species (ROS) to facilitate adaptation in abiotic stress environments. A SNP (C/T) within the *BM* coding sequence causes this variation. The beauty mark and UV floral patterns are associated phenotypes. UV exposure resulted in increased ROS generation in cotton floral tissues. Hence, by exploiting allelic variation associated with *GbBM*, cotton cultivars with enhanced pollinator activity, resistance to UV-exposure, and tolerance to abiotic stress could be developed.

#### Floral reward

**Table 5** lists genomic regions (QTL) and/or putative genes associated with floral reward traits. Major QTL and candidate genes, including *HaCWINV2* associated with nectar volume and/or production, are detected in sunflower (Barstow et al., 2022, 2025). The increased nectar volume indicates a greater quantity of sugars and total energy per floret available to pollinators. The candidate genes underlying QTL associated with nectar volume are homologous to genes with nectary function in *Arabidopsis* (Barstow et al., 2022). *BLADE-ON-PETIOLE*-like genes in pea and *Arabidopsis* regulate floral nectary development, while *CYCLOIDEA*-mediated adaxial developmental program suppresses floral nectary development in other legumes (Sinjushin, 2022). Squash (*Cucurbita pepo*) is a perfect model for studying nectar biology. It has large-sized nectaries that produce a substantial amount of nectar. Comparative metabolic profiling of nectaries, involving male and female flowers on the same plant, shows progression from starch synthesis to starch degradation and to sucrose biosynthesis. A sucrose-rich nectar is therefore synthesized and secreted. Forty metabolites were detected in both male and female flowers, of which some display preferential accumulation and differentially expressed genes in the nectar of either male or female flowers (Solhaug et al., 2019a). The genetic model for nectar secretion in *Arabidopsis* matches that of squash. In addition, the sugars imported from the phloem during nectar secretion are equally important for generating nectar in squash. The phloem sugar, however, is not stored as starch in the nectary as is the case for *Arabidopsis*. Trehalose and trehalose 6-P regulate nectary starch degradation and nectar secretion in squash (Solhaug et al., 2019b). Transcriptome profiling of pepper (*Capsicum annuum*) flower at three development stages revealed many differentially expressed genes (DEGs) and pathways associated with nectar biosynthesis and nectary development. DEGs were involved in various metabolic processes, including flower and nectary development and nectar biosynthesis. The sucrose-phosphatase, galactinol-sucrose galactosyltransferase, and sucrose synthase regulated nectar biosynthesis, while *CRABS CLAW* is potentially involved in mediating nectary development (Deng et al., 2020).

**Table 5:** Quantitative trait loci (QTL) and candidate genes associated with floral reward traits in *Arabidopsis* and diverse crops.

Candidate gene	Floral reward traits	Reference
Grain crop		
*Pisum*		
*BLADE-ON-PETIOLE*-like genes *CYCLOIDEA*-mediated adaxial developmental program	Regulate floral nectary development between *Pisum* and *Arabidopsis* Suppresses floral nectary development in other legumes	Sinjushin, 2022
Oil crop		
Mustard (*Brassica rapa*)		
*BrCWINV4A*	*brcwinv4a* mutant produces sucrose-rich nectar, but significantly less in volume	Minami et al., 2021
*SWEET9*	Nectar production and secretion	Lin et al., 2014
	Sunflower (*Helianthus annuus*)	
*HaCWINV2* (9 putative homologs of Arabidopsis genes with nectary function within QTL regions	Nectar production (PVE 48.55%)	Barstow et al., 2025
Major QTL mapped on chr. 2 and 16	Nectar volume	Barstow et al., 2022
Vegetable crop		
Bell pepper (*Capsicum annuum*)		
DEGs associated with nectar biosynthesis and nectary development: B2 vs B1: 8,955; B3 vs B1: 12,182; B3 vs. B2: 23,667, involved in flower development, nectar biosynthesis, and nectary development	Sucrose-phosphatase, galactinol-sucrose galactosyltransferase, and sucrose synthase regulate nectar biosynthesis, while *CRABS CLAW* mediated in nectary development	Deng et al., 2020
Squash (*Cucurbita pepo*)		
Trehalose and trehalose 6-P regulate starch degradation and nectar secretion in nectary	Starch degradation and nectar secretion	Solhaug et al., 2019a, b
Ornamental crop		
*Cleome violacea*		
*CvCRC*, *CvAG, CvSHP, CvSWEET9*, and uncharacterized transcript	*CvCRC*, redundantly with *CvAG* and *CvSHP*, regulate nectary initiation *CvSWEET9* essential for nectar formation and secretion	Carey et al., 2023
*Liriodendron tulipifera* (Tulip tree)		
56 TFs, including *TCP*, *Trihelix*, *C2H2*, *ERF*, and *MADS* families	Nectary development and nectar secretion	Liu et al., 2019
*Penstemon barbatus* (Golden beared-penstemon)		
Nectaries develop independently of the canonical core-eudicot CRABS CLAW genetic module, involved in *Arabidopsis* and *Petunia*	Nectar production follows conserved sugar metabolic pathways	
*Petunia hybrida* (Petunia)		
*EOB1 and EOB2*	regulate flower maturation and senescence or floral scent emission	Chopy et al., 2023
*EOB1 and EOB2*	Control floral scent emission	
C-lineage genes (*euAG* and *PLE*) trigger nectary development; TARGET OF EAT-type *BLIND ENHANCER* and APETALA2-type *REPRESSOR OF B-FUNCTION* regulates nectary size	Carpel-associated nectary development	Morel et al., 2018

Major QTL or candidate genes associated with floral display traits in ornamental/fragrant crops, for example, major QTL associated with flower color variation in *Chrysanthemum* and *Petunia*; floral petal depth and diameter in *Petunia*; anthocyanin and carotenoid contents in *Ch*rysanthemum flowers; and petal elongation (including ray petal) in gerbera daisy (*Gerberas hybrida*) (**Table 4**). Genes regulating carpel-associated floral nectary development and size in *Petunia* and *tarSWEET 9*, *tarCWIN4*, *tarCWIN6*, and *tarSPAS2* regulating nectar secretion in response to pollination cues in dandelion species (*Taraxacum* spp.) (**Table 5**). The putative (or candidate) genes must be functionally validated across genetic backgrounds and environments before their use in plant breeding programs.

### Crop domestication, polyploidy, mating systems, and inbreeding effects on plant-pollinator interaction

#### Crop domestication

Domestication reduces genetic diversity, often making crops more susceptible to pests and diseases. Did domestication alter plant-pollinator interactions affecting pollinators’ health? Pollinator-favored floral display and reward (i.e., flower size, shape, and color; nectar volume and composition; pollen nutritional quality) traits have direct bearing on pollinator visitation and foraging decisions by altering the chemicals and pollen, potentially affecting pollinator health via changes in pathogens. Domestication has significantly altered the chemical composition of highland blueberry (*Vaccinium corymbosum*) nectar and pollen, as well as reduced pollen chemical diversity. Thirteen of the 20 metabolites differ significantly between wild and cultivated plants, with the most positively associated in the wild blueberry plants. The phenylalanine, a known bee phagostimulant (a substance that increases the palatability of food for bees) and essential nutrient, was 4.5 times higher in wild nectar and 11 times higher in wild pollen, while caffeic acid ester (an antimicrobial compound) was two times higher in wild nectar. Caffeic acid is associated with reduced bumblebee (*Bombus impatiens*) infection by *Crithidia* (a prominent gut pathogen) at concentrations detected in wild but not in cultivated blueberry, thus suggesting domestication influences floral traits with consequences for bee health (Egan et al., 2018). However, it should be noted that metabolite concentrations may vary across genetic backgrounds and environments, necessitating the need for multi-site evaluation, and this variation may influence pollination, herbivory, and disease in wild and domesticated plants. The domesticated pumpkin (*C. moschata*, *C. argyrosperma* ssp. *argyrosperma*) and its wild progenitor (*C. argyrosperma* ssp. *sororia*) experienced different selection pressures. The domesticated species invested more resources in floral traits (i.e., larger staminate (male) and pistillate (female) flowers; greater pollen quantity and protein to lipid ratio), thereby increasing attractiveness to cucurbit-pollen specific pollinators (*Eucera* spp) for reproductive success (Glasser et al., 2023). More recently, it was further demonstrated, by including three domesticated and three wild *Cucurbita* species, that domesticated *Cucurbita* species exhibit larger floral morphological traits in both pistillate and staminate flowers compared to their wild relatives. The nectar volume increased in domesticated species, while sugar and amino acid concentrations remained unchanged. Contrarily, domestication had no significant effect on pollen production, pollen size, and pollen protein or lipid contents. This suggests that domestication differentially affects floral traits (i.e., while floral morphology is significantly altered, most of the floral reward traits remain largely unaffected). It seems a complex interplay between domestication, resource allocation, and plant–pollinator interactions shape floral traits in pumpkin (Villanueva‐Espino et al., 2025).

Domestication also mediates plant-pollinator interactions as evidenced in pumpkins (*Cucurbita*), a genus with multiple wild and cultivated species. The floral volatile organic compounds (VOC) signal the presence of quality floral resources for pollinators (Magalhães et al., 2025; Ye et al., 2025). The characterization of wild and domesticated *Cucurbita* species revealed that domesticated plants (*C. pepo* ssp. *ovifera* var. *ovifera*), relative to wild species, had significantly lower floral VOC richness. A total of 10 physiologically active compounds were detected across wild and domesticated squash VOCs, with 1,4-dimethoxybenzene as being the most dominant volatile in domesticated squash blends, while (E)-nerolidol was abundant in most wild species except *C. foetidissima* and *C. maxima* ssp. *andrean*a. The increased bee visitation was significantly associated with increased emissions of 1,4-dimethoxybenzene, dihydro-β-ionone, and (*E*)-nerolidol, and reduced emissions of linalool and methyl salicylate (Singh et al., 2025).

#### Hybridization, polyploidy, and mating system

Hybridization and variation in polyploidy levels have the potential to change floral traits affecting pollinators’ visit and foraging behavior. Mating system differences may interact with ploidy to affect floral traits and pollination niches. Hybrid flowers may exhibit new trait combinations, e.g. from petal color to nectar composition or variation in corolla size, which could attract new pollinator species. Hybrids between a hummingbird (*Trochilidae* family and native to the American continent)-pollinated species *Mimulus cardinalis* and a self-pollinated species *M. parishii* attracted bumblebees (*Bombus impatiens*), a pollinator not attracted to either of the progenitor species. Bees readily discriminate flower color variation, with a large portion of this variability controlled by *YUP* locus in *Mimulus* species. Thus, attraction of new pollinators to hybrid plants may provide new avenues for pollinator shift and speciation (Peng et al., 2024).

Assessment of diploids and synthetic tetraploids of *Arabidopsis arenosa* on pollinator behavior revealed no significant differences between diploid and tetraploid plants. The tetraploids were, however, more productive than diploids, thereby indicating the beneficial effect of polyploidization on pollen transfer via other genetic means. Polyploidization promoted interploidy pollen exchange as the pollinators preferentially switched between cytotypes (diploid, triploid or tetraploid) rather than preferring one cytotype (Schmickl et al., 2024). A study investigating the impact of polyploidy and mating system on floral traits and pollination niches in *Brassica* species revealed that flower size, but not shape, depended on the interaction between polyploidy and mating system. The self-incompatible polyploid species had larger flowers than self-incompatible diploids, while no such differences in the size of the flowers were observed among self-compatible species. The pollination niche (or ecological niche associated with pollination) was not affected by ploidy. It was, however, strongly affected by differences in mating systems (Streher et al., 2024).

#### Inbreeding

Inbreeding refers to the mating of individuals that are related by ancestry and is accompanied by inferior offspring performance. A study involving inbred and outbred maternal families of horsenettle (*Solanum carolinense*) revealed that flowers of inbred plants exhibited reduced corolla size and pollen production, and significantly reduced emission of volatile compounds. Bumblebees (*Bombus* spp.), the main pollinators of horsenettle, discriminated against inbred and outbred flowers in the field. The bees preferred visiting more frequently and spending more time in outbred flowers. Thus, inbreeding alters floral traits, reduces floral rewards (nectar volume and pollen grains), and impacts (adverse) pollinator visitation and foraging behavior (Kariyat et al., 2021). An earlier report involving monkey flower (*M. guttatus*) plants and bumblebee (*B. impatiens*) pollinators in a greenhouse also showed that inbreeding (relative to outbreeding) had reduced resource quality for pollinators, adversely impacting pollinator populations (Carr et al., 2014).

### Floral volatiles and secondary metabolites in pollinator signaling

**Figure 2:**
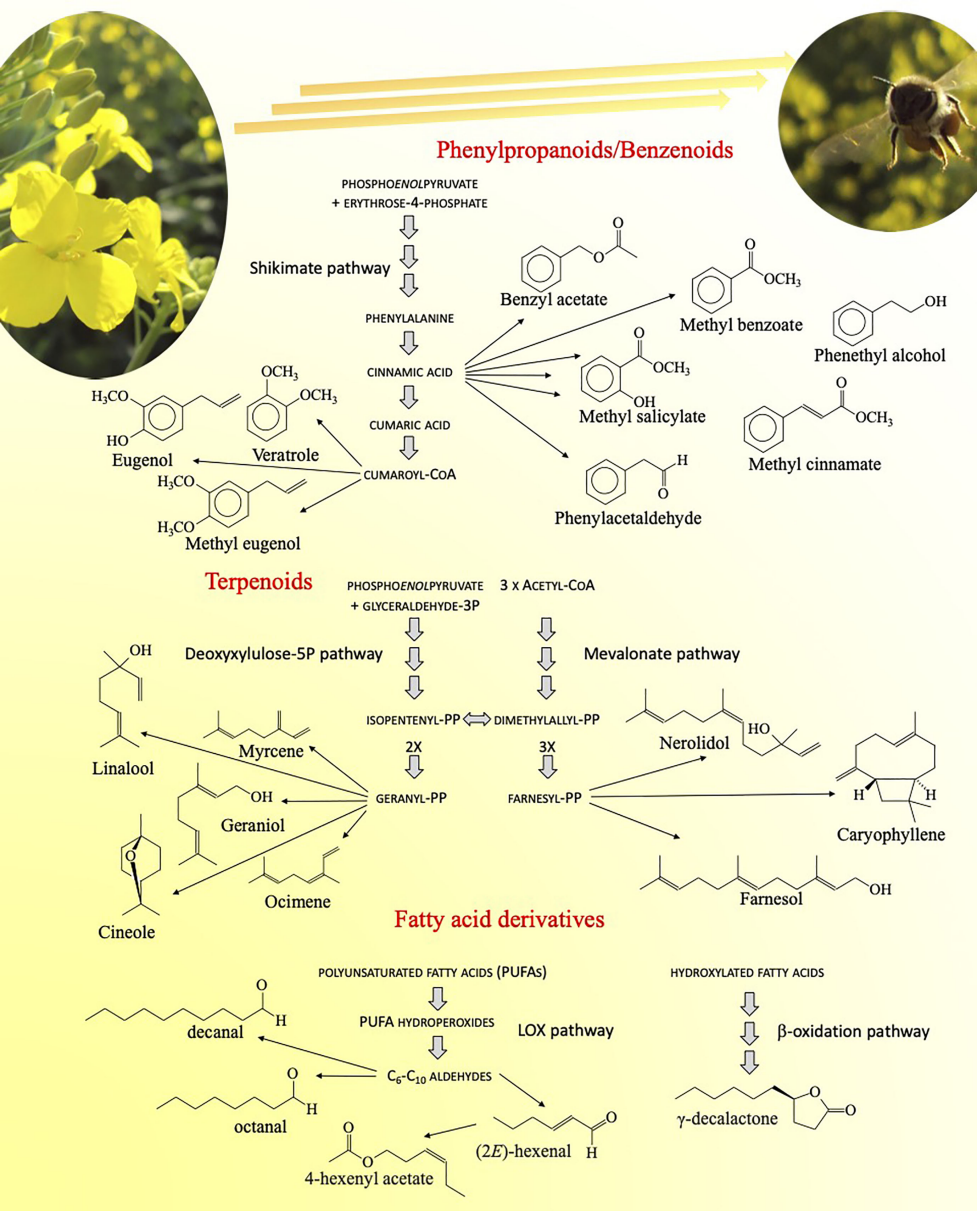
Volatile organic compounds produced by flowers to attract pollinators, and the metabolic routes leading to their biosynthesis. These substances belong to different groups of secondary metabolites, namely phenolics, terpenes and fatty acid derivatives.

Besides the relevance for the floricultural and perfume industry, floral scent plays a crucial role in attracting pollinators (Dötterl and Gershenzon, 2023). Fragrance is determined by the production of floral volatiles (Lo et al., 2024), a complex mixture of secondary metabolites that belong to three major groups: fatty acid derivatives, phenylpropanoids/benzenoids (Lv et al., 2024), and terpenoids (Boncan et al., 2020) (**Figure 2**). Many fragrance elements are highly conserved and produced in most plant species, but the blend is unique. Like most visual cues, such as flower color, shape, and pattern, these volatiles are mainly synthesized in petals. Consistently, the genes involved exhibit specific or high expression in epidermal cells of petals. However, despite significant advances in identifying these genes (Chen and Liao, 2025; Mostafa et al., 2022; Pichersky, 2023), little is known about their dynamic expression in a given plant species, leading to its unique blend.

A recent study provided a first look into the pathways driving floral scent biosynthesis. Using single-cell RNA sequencing, gene expression was dissected in petals during four developmental stages in Chinese plum (*Prunus mume*). Six distinct cell types were identified, and 28 genes in the benzenoid/phenylpropanoid pathway showed maximal expression in coincidence with the peak production of the two main volatiles, benzyl acetate and eugenol (Guo et al., 2024). The obtained single-cell atlas offers a comprehensive view of the molecular mechanisms underlying fragrance production, opening new perspectives to study pollinator preferences. Another study shed some light on the genetic mechanisms underlying volatile production in *Petunia*. The homeotic gene *DEF*, besides being responsible for petal identity, was found to initiate the synthesis of scent phenylpropanoids by activating two MYB transcriptional regulators, *EMISSION OF BENZENOIDS I* and *II* (Bednarczyk et al., 2025). The increasing availability of RNA sequencing and heatmapping techniques is leading the path toward the identification of all genes involved in volatile production, and the measurement of their differential expression to provide a given scent. This approach has been described in a study on the Oriental lily ‘Sorbonne’, where key genes and transcription factors have been identified that are associated with the production of a number of monoterpene volatiles (Cao et al., 2025). Powerful omics approaches will also be required in view of the ongoing climate change, as the synthesis of floral volatiles has been found to be affected by abiotic stress conditions. When plants of the sub-alpine species *Ipomopsis aggregata* were subjected to increasing drought, volatile release increased overall and changed in composition, with a decrease of the monoterpene α-pinene and an increase of the sesquiterpene α-farnesene (Campbell et al., 2019). The emission of scent volatiles by flowers of three *Brassicaceae* subjected to a combination of progressive water deficit and nitrogen supplementation varied both quantitatively and qualitatively, with terpenoid synthesis the most negatively affected by drought (Höfer et al., 2022). Flower storage at low temperatures led to the loss of rose fragrance because of epigenetic downregulation of scent-related genes, such as germacrene synthase and phenylacetaldehyde reductase (Xie et al., 2023). A shift of 5 °C over the optimal growth temperature was found to completely prevent the emission of floral volatiles by strawberry plants, which under optimal conditions released 10.4 ng *per* flower *per* hour of a mixture of eight scent compounds, mainly *p*-anisaldehyde (Cordeiro and Dötterl, 2023). Water and temperature stress conditions are therefore susceptible to impact on plant-pollinator interactions that are mediated by floral volatiles.

Plants rely on volatile organic compounds to attract pollinators, while synthesizing a nectar rich in sugars and amino acids to reward them and ensure their fidelity. In recent years, the idea of a nectar merely representing a recompense for help in pollination has been superseded. Floral nectar must be considered an interface by which plants maintain complex interactions with other organisms. This new perspective came from the identification in nectar of several secondary metabolites. Increasing evidence suggests that amino acid content plays a main role in pollinator preference and fidelity (Broadhead and Raguso, 2021; Bouchebti and Levin, 2024; Carlesso et al., 2021; Parkinson et al., 2025). On the other hand, secondary metabolites interact with pollinators’ nervous system and condition their behavior (Nicolson, 2022), protect nectar itself from saprophytic microorganisms (Schmitt et al., 2021) and robbers (Barberis et al., 2023), and in some cases promote pollinator health (Slavković and Bendahmane, 2023). Being a high-energy, sugary liquid, nectar can support the growth of microbial communities often conveyed by the pollinators themselves (Morris et al., 2020; Jacquemyn et al., 2021) that, even if benign to the plant, significantly alter its content. Shifts in nectar microbial communities have been found because of extreme heat, suggesting that climate change might indirectly influence nectar composition (Russell and McFrederick, 2022). Such modifications of nectar chemistry are susceptible to influence in turn pollinator behavior, thereby decreasing visitation frequency (Vannette et al., 2012; Quevedo-Caraballo et al., 2025). The production and the release into the nectar of secondary metabolites endowed with antimicrobial activity can therefore avoid unwanted/excessive microbial growth (Schmitt et al., 2021; Xun et al., 2025).

Nectar components derive mainly from the phloem, but their concentration is influenced by nectaries metabolism. To date, our knowledge of plant secondary metabolites in nectar is still limited, since most metabolomics studies exclusively reported on sugars and amino acids, and only a few transcriptomic analyses have been performed with nectaries. A metabolic and transcriptomic analysis of nectaries recently revealed differences in the mechanism of nectar production between monocots and dicots (Göttlinger et al., 2024), but in this case also, the focus was mainly on sugar metabolism and transport. A deeper and systematic analysis of secondary metabolite content is needed –in view of a better understanding of their effect on pollinators– because nectar composition may also be significantly affected by abiotic stress conditions. In an epiphytic bromeliad species, nectar volume and composition were found to be unaffected by different temperatures or varied light conditions, but strongly affected by drought. Water stress strongly decreased both nectar volume and its sucrose-to-hexose ratio, despite no changes being evident in the nectaries (Göttlinger and Lohaus, 2020). In another study on four *Nicotiana* species, either day- or night-flowering, nectar volume was found to be affected by drought with a reduction that was proportional to the severity of the stress. Nectar concentration only mildly increased as a consequence, with a resulting proportional decrease of sugars, amino acids, and inorganic ions *per* flower. Also in this case, with the only exception of *Nicotiana otophora*, the sucrose-to-hexoses ratio showed a striking lowering, suggesting specific effects on nectary metabolism (Göttlinger et al., 2025). *Zm*SWEET1b, a member of the sugar efflux transporter SWEET family that is involved in multiple biological processes, among which nectar secretion, was consistently found to be involved in the maize plant response to salt stress (Wu et al., 2023).

### Trade-offs involving reproductive success, florivory, and pollinator attraction

Complex genetic mechanisms governing flower development and function are involved in the molecular basis of trade-offs between reproductive and pollinator-relevant floral traits (Filipiak et al., 2022). These trade-offs arise from the dual role of floral traits as both reproductive structures and pollinator attractants (Balfour et al., 2025; Pattrick et al., 2023; Van der Niet et al., 2014), thereby bringing inherent conflicts in resource allocation and structural optimization. Genes controlling floral traits face competing selective pressures. Different pollinators vary in their perception of floral attractant cues and morphological requirements, thus leading to divergent selection on floral characteristics (Van der Niet et al., 2014). For example, flowers adapted to specific pollinators often show enhanced characteristics improving their fit with flower reproductive organs. This specialization can, however, compromise effectiveness with other pollinators. Furthermore, as shown by Lanuza et al. (2021), plant reproductive trade-offs determine plant-pollinator interactions as well as plant life history strategy.

The Geographical Mosaic Theory (Gomulkiewicz et al., 2007) explains how these trade-offs manifest across different environments. As plant populations adapt to increase efficiency with local pollinators, they often face a “cost” in the form of reduced attraction or efficiency toward their original pollinator groups (Van der Niet et al., 2014). This divergent selection drives the formation of pollination ecotypes, in which floral traits—such as morphology, color, and scent—evolve to match the preferences of local pollinator communities. Ultimately, these molecular and phenotypic adaptations must balance the competing demands of maximizing pollinator attraction while ensuring overall reproductive success.

Moonflower (*Rivea ornata*) is known for its impressive white or pale-yellow flowers. The flowers attract both pollinators as well as florivores (i.e., nocturnal hawk moths [*Sphinx ligustri*] that primarily depend on flowers for their diets), compromising reproductive success. It is a self-incompatible and obligate outcrossing species, thereby highly dependent on pollinators. Florivory, however, does not significantly reduce its reproductive success. How does moonflower achieve a balance between florivory and pollinators? The plant invests more resources in defending key floral structures while guarding flowers from ants’ damage, resulting in minor florivore damage to non-vital floral organs. Thus, it ensures that pollinators are not deterred by ants and maintains high pollinator visitation rates (Chitchak et al., 2024).

Understanding these molecular trade-offs, as indicated by Armbruster (2014), is crucial for describing plant speciation processes, as they can bring reproductive isolation when populations become adapted to different pollinator regimes. This connection between molecular-level trait evolution and species divergence demonstrates also how seemingly minor modifications in floral traits can cascade into significant evolutionary outcomes.

### Breeding and biotechnology to enhance pollination: opportunities and constraints

#### Crossbreeding

Many floral resource traits (e.g., flower size and shape, color, or attractiveness) are interrelated (Brunet et al., 2021), with moderate to high heritability, and controlled by few genes (Barstow et al., 2022). A few functionally characterized genes and many QTL or candidate genes associated with floral resources, including nectar volume and sugar in the nectar, have been unlocked. Harnessing variability favoring plant-pollinator interactions, along with other beneficial traits (e.g., stress tolerance and edible yield attributing genes), through crossbreeding and selection, poses a significant breeding challenge but may provide new avenues to breed climate-resilient and pollinator-friendly crops for sustainable farming systems. However, a potential trade-off involving resource allocation between reproduction and floral structural optimization (to attract pollinators) or between reproduction and herbivory and/or abiotic stress tolerance may pose a significant biological constraint to combining these competitive traits in new germplasm. Deploying optimal cross combinations and multi-trait genomic selection in crop breeding could facilitate the development of such crop germplasm (Alemu et al., 2024; Miller et al., 2023).

The genomes of several insect-pollinated model plants like petunia (Saei et al., 2024) and monkey flower (*Mimulus guttatus*; Hellsten et al., 2013) and field crops such as faba bean (Jayakodi et al., 2023; Zhang et al., 2024), rapeseed (Song et al., 2020) and mustard (Perumal et al., 2020), sunflower (Badouin et al., 2017; Yi et al., 2025), tobacco (Wang et al., 2024b), and tomato (The Tomato Genome Consortium, 2012), have been sequenced which may guide us to unlock molecular basis of plant-pollinator interactions. Rapid advances in sequencing technologies and a reduction in sequencing cost, such as genotype-by-sequencing (GBS), have facilitated SNP discovery in several plant species. Hence, GBS is a quick and affordable reduced representation method to simultaneously identify and discover many SNPs in a wide range of plant species (Pootakham, 2023). Accessing and exploiting functional diversity for plant-pollinator interactions, identifying genomic regions and candidate genes associated with floral resources, followed by functional validation of such genes and markers, and deploying them in breeding programs, should facilitate the development of climate-resilient and pollinator-friendly crops. For example, SNP markers associated with the cell wall invertase gene, *CWINV2*, can be deployed to increase sucrose concentration in sunflower inbreds and hybrid cultivars (Prasfika et al., 2018). Sucrose concentration in the nectar influences bee foraging in sunflowers. Floret size is another interesting trait, as small changes in floret size (reducing corolla depth) result in dramatic effects on nectar access by wild bees (Portlas et al., 2018). A highly reproducible SNP marker tightly linked to the causal gene (*CmCCD4a*) associated with petal (ray florets) color development may be deployed in breeding programs to develop new chrysanthemum germplasm with enhanced plant-pollinator interactions (Sumitomo et al., 2019). Cultivated chrysanthemum is a hexaploid species with hexasomic inheritance (van Geest et al., 2017).

Flower resources (flower number, size and shape of flowers, nectar, nutritional quality of pollen, and scent in case of fragrant crops) impact bees’ visitation. Assessing which flower trait impacts bees’ visit and foraging behavior is a significant challenge. Using enclosed arenas (custom-built flight arenas of 90 × 54 × 46 cm), two solitary bee species (*Osmia cornifrons* and *Megachile rotundata*) and recombinant inbred lines (originating from a cross involving a self-fertilizing *Capsella rubella* and the pollinator-dependent outcrosser *C. grandiflora*) segregating for a range of floral traits, it was demonstrated that *O. cornifrons* visits were positively correlated with the number of flowers, while *M. rotundata* visits were associated with pollen nutrition, especially for plants with a higher pollen protein-to-lipid ratio. Further investigation involving artificial flowers with liquid diets (Kraus et al., 2019; Wright et al., 2018) confirmed that *M. rotundata* preferred flowers with higher protein-to-lipid ratios, while *O. cornifrons* visits were unaffected by pollen nutrition, thus suggesting that bee species differentially prioritize floral resources (Mokkapati et al., 2024).

#### Biotechnological interventions

**Figure 3:**
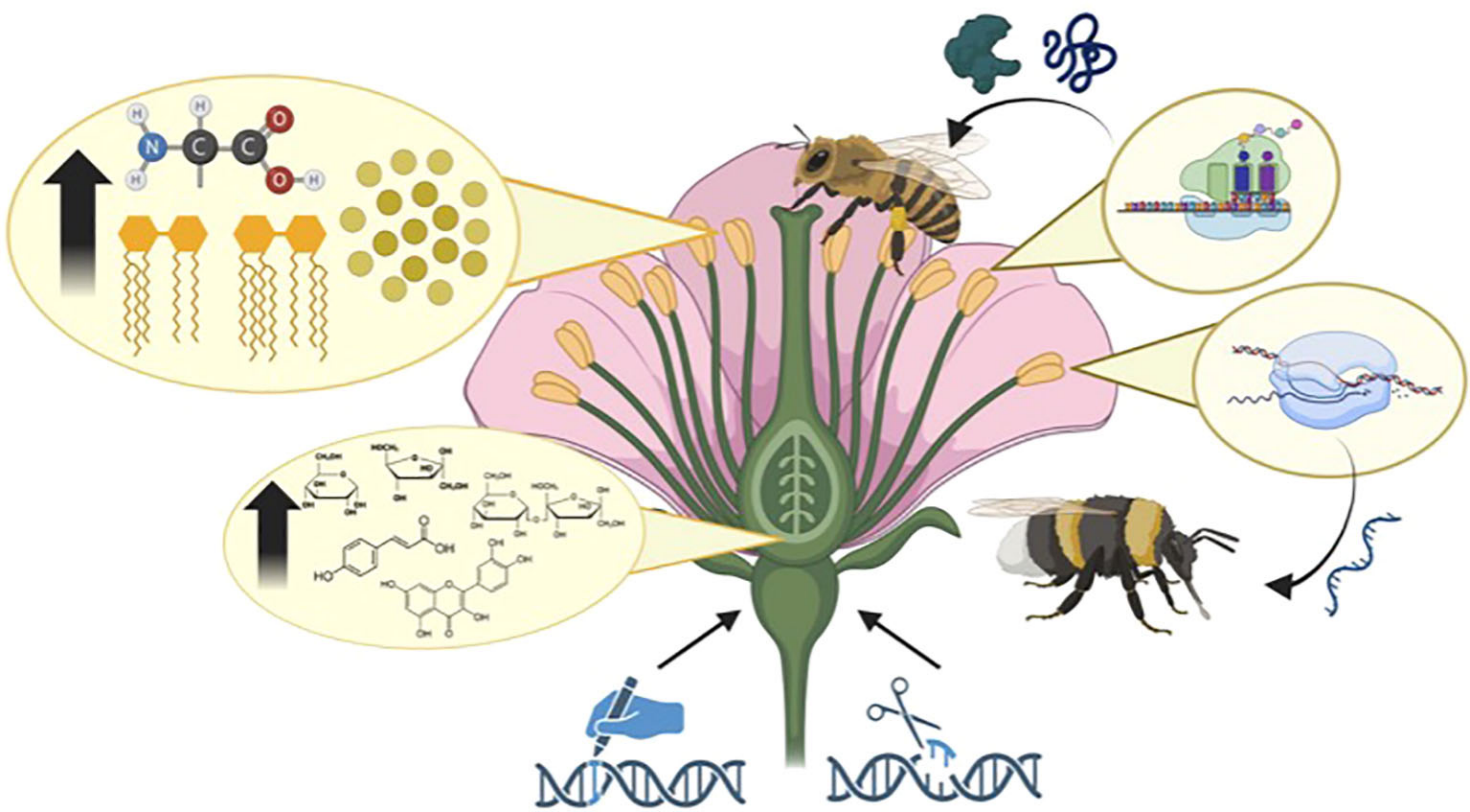
Targeted genetic modifications to floral structures—specifically pollen and nectar–can be leveraged to benefit pollinators through improved nutrition and therapeutic delivery. Pollen can be metabolically engineered to increase its protein and lipid content, with a focus on essential amino acids and fatty acids that are critical for pollinator development, immune function, and longevity. In parallel, pollen-producing tissues can be used as platforms for the *in planta* synthesis of functional RNAs. These RNA molecules, including double-stranded RNA for RNA interference (RNAi) or other gene regulatory RNAs, could be delivered orally to pollinators as a form of biocontrol or gene therapy, targeting pathogens, parasites, or enhancing pollinator resilience. Nectar traits can also be enhanced through metabolic engineering to increase sugar concentration, improving its energetic value and potentially attracting more frequent pollinator visits. Modifying nectar production could also help sustain pollinator foraging during periods of floral scarcity or environmental stress. Two key genetic engineering approaches are depicted. CRISPR-based gene editing enables precise modifications of endogenous genes involved in floral metabolism and biosynthetic pathways. Transgenesis, by contrast, allows for the introduction of entirely novel genes or synthetic regulatory circuits to endow plants with new pollinator-supportive traits. Together, these could enable the engineering of flowers that actively contribute to pollinator health and the stability of plant–pollinator ecosystems.

Biotechnology provides powerful tools to enhance plant characteristics specifically aimed at improving pollinator health (**Figure 3**). Enhancing the nutritional quality of nectar and pollen via metabolic engineering is one route to enhance pollinator food resources. Nectar serves as a primary energy source for pollinators, especially bees, hummingbirds, and butterflies. Key biosynthetic pathways in plants could be adjusted to enrich nectar composition. Several transcription factors have been identified as regulators of nectar biosynthesis, influencing floral nectary development and metabolic activity (Slavković et al., 2021). Similarly, modulating pollen nutrition profiles by increasing protein and lipid content could improve pollinator health and reproduction.

Biotechnology also enables the manipulation of floral volatiles (Pichersky and Dudareva, 2007), which could be used to better attract pollinators. Floral scent is critical for attraction and navigation, serving as chemical cues guiding pollinators to flowers (Burkle and Runyon, 2017). Transgenic approaches have successfully been used to modulate floral scent through metabolic engineering of terpenoid and phenylpropanoid biosynthetic pathways (Kessler et al., 2008). Such modifications could enable tailored fragrance profiles, to increase pollination efficiency and support pollinator health by increasing resource availability. Optimizing floral scents could also aid pollinator navigation in fragmented or degraded habitats, effectively increasing their foraging efficiency and reducing energetic stress (Burkle and Runyon, 2017).

Biotechnological interventions can improve plant resistance to pathogens and pests, which indirectly benefits pollinators by reducing agrochemical use. Engineered plants expressing resistance traits to common diseases or herbivory typically require significantly fewer pesticide applications (Brookes, 2022). For instance, transgenic plants expressing *Bacillus thuringiensis* (Bt) toxins selectively target pests, while leaving pollinators unharmed, resulting in reduced pesticide use and exposures (Duan et al., 2008 Yaqoob et al., 2016). Similarly, plants engineered for resistance to fungal pathogens could decrease the need for fungicides, thus limiting off-target effects on pollinators.

The emerging field of synthetic biology (SynBio) provides additional opportunities to augment plant-pollinator interactions. SynBio enables more complex, multigenic trait modifications that could prove useful in the development of cultivars with synchronized blooming cycles or extended flowering periods. Such traits will be important for ensuring consistent and reliable food sources for pollinators, particularly under shifting climatic conditions (Wang et al., 2024).

CRISPR-based approaches have enabled precise manipulation of flowering regulatory genes (Bellec et al., 2022; Li et al., 2021; Shang et al., 2024). Such approaches could be used to produce plant lines with a prolonged bloom duration or synchronized flowering events optimized to match pollinator activity. This synchronization would mitigate temporal mismatches driven by climate change, ensuring stable resource availability throughout critical periods in pollinator life cycles.

Despite these promising applications, biotechnological interventions in plant–pollinator systems must be evaluated within an ecological and socio-economic context. Metabolic modifications intended to enhance nectar or pollen quality could alter plant resource allocation, potentially affecting growth, stress tolerance, or interactions with non-target organisms. Changes in floral scent profiles or bloom timing may have unintended consequences for plant reproductive success, community dynamics, or specialized pollinators adapted to native signaling cues. Engineered resistance traits, while reducing pesticide use, also raise concerns regarding resistance evolution in pests and potential indirect ecological effects. Additionally, public acceptance, regulatory complexity, gene flow to wild relatives, and the need for long-term ecological monitoring represent practical limitations to widespread implementation. A precautionary, systems-level approach that integrates molecular innovation with ecological risk assessment will therefore be essential to ensure that plant biotechnology advances pollinator health without generating unintended tradeoffs.

## Conclusion and outlook

Anthropological changes in recent decades have reduced the abundance and density of pollinator diversity, resulting in a global pollinator crisis that may threaten food and nutritional security, especially in the Global South. While major food staples like wheat and rice have been inbred for centuries—bypassing the need for pollinators—they remain vital to the biological landscape. By providing consistent floral resources and stable habitats, these crops serve as essential refuges for pollinators during times of scarcity. Their presence ultimately strengthens the ecological web, supporting ecosystem services like soil stabilization and nutrient cycling that extend well beyond their own reproductive cycles.

The integration of DARkWIN platform with other high-throughput phenotyping methods may bridge the gap between genotype expression and ecological function. Pollinator-dependent crops show sufficient natural variability for floral display (size, shape, color, and attractiveness) and floral reward (nectar, pollen) traits, controlled by multiple genes. The domestication and inbreeding negatively affect the plant-pollinator interactions, while hybridization, polyploidy, and mating systems have the potential to change floral display and reward traits for better expression, hence attracting more pollinators to visit and forage on flowers, promoting pollination. The genetic tradeoffs involving reproductive and pollinator-relevant floral traits should be factored in while developing strategies for pollinator-friendly crops.

Research suggests that it is feasible to integrate pollinator-friendly traits together with stress tolerance, yield, and nutrition traits following crossbreeding and biotechnological interventions in ornamental and field crops. The focus should be on (i) assessing and exploiting functional diversity in floral-display and floral-reward traits to facilitate pollinator visitation, (ii) unlocking the molecular basis of tradeoff involving resource allocation between competitive traits (reproduction, floral structural rearrangement favoring pollinators, herbivory or abiotic stress adaptation), (iii) integrating advances in crossbreeding and biotechnology applications to develop crop germplasm promoting plant-pollinator interactions, (iv) sequencing pollinator genomes to identify sequence differences and candidate genes associated with pollinators preferences for visitation and foraging of plant floral organs, (v) promoting pollinator-friendly practices or growing ornamental crops in the vicinity of field crops to promote plant-pollinator interactions, (vi) identifying pollinator and agrobiodiversity hotspots across regions and establish *ex situ* conservation of native pollinators as an entrepreneurial opportunity like beekeeping enterprises, (vii) organizing regular awareness and training campaigns advocating for pollinator‐friendly initiatives, and (viii) promoting collaboration with multiple stakeholders, including researchers, gardeners, farmers, conservation biologists and ecologists, and policymakers, is a prerequisite to sustain agro-biodiversity (including pollinators) conservation, and restore pollination services to enhance food and nutritional security. All of them are crucial for enhancing plant-pollinator interactions to ensure food and nutritional security.
